# Disruption of Daily Rhythms by High-Fat Diet Is Reversible

**DOI:** 10.1371/journal.pone.0137970

**Published:** 2015-09-14

**Authors:** Katrina L. Branecky, Kevin D. Niswender, Julie S. Pendergast

**Affiliations:** 1 Department of Medicine, Division of Diabetes, Endocrinology and Metabolism, Vanderbilt University School of Medicine, Nashville, Tennessee, United States of America; 2 VA Tennessee Valley Healthcare System, Nashville, Tennessee, United States of America; McGill University, CANADA

## Abstract

In mammals a network of circadian clocks coordinates behavior and physiology with 24-h environmental cycles. Consumption of high-fat diet disrupts this temporal coordination by advancing the phase of the liver molecular clock and altering daily rhythms of eating behavior and locomotor activity. In this study we sought to determine whether these effects of high-fat diet on circadian rhythms were reversible. We chronically fed mice high-fat diet and then returned them to low-fat chow diet. We found that the phase of the liver PERIOD2::LUCIFERASE rhythm was advanced (by 4h) and the daily rhythms of eating behavior and locomotor activity were altered for the duration of chronic high-fat diet feeding. Upon diet reversal, the eating behavior rhythm was rapidly reversed (within 2 days) and the phase of the liver clock was restored by 7 days of diet reversal. In contrast, the daily pattern of locomotor activity was not restored even after 2 weeks of diet reversal. Thus, while the circadian system is sensitive to changes in the macronutrient composition of food, the eating behavior rhythm and liver circadian clock are specifically tuned to respond to changes in diet.

## Introduction

Circadian rhythms are ~24-hour rhythms of physiology and behavior that are synchronized to environmental cycles of light/dark and fasting/feeding [1]. Circadian rhythms in mammals are orchestrated by a network of clocks located throughout the body. The suprachiasmatic nucleus (SCN) in the hypothalamus receives information about the light-dark cycle and coordinates the phases of other central and peripheral clocks [2, 3]. Disruption of the coordinated phase relationship between these clocks in rodents by chronic jet lag and shift work causes obesity [4, 5]. Likewise, shift workers are at increased risk of obesity, the metabolic syndrome, and cardiovascular disease [6–10].

We recently demonstrated that eating a diet high in saturated fat for only 1 week disrupts the phase relationship between circadian clocks in male mice by advancing the phase of the liver clock by ~5h [11]. Moreover, high-fat diet markedly reduces the amplitude of the daily rhythm of eating behavior such that eating behavior is almost equally distributed across the day and night [11, 12]. This low-amplitude eating rhythm during high-fat diet consumption is a determinant of obesity because correcting the rhythm (by restricting high-fat diet to the nighttime) inhibits diet-induced weight gain [13, 14].

Studies in mice have demonstrated that diet-induced obesity and type 2 diabetes (hyperglycemia) caused by chronic high-fat diet consumption are partially or completely reversed by feeding mice low-fat diet [15–17]. In this study, we sought to determine whether the effects of chronic high-fat diet consumption on circadian rhythms were ameliorated by diet reversal.

## Materials and Methods

### Animals

Male C57BL/6J heterozygous PERIOD2::LUCIFERASE mice [3] (23 to 24 generations of backcrossing to C57BL/6J mice from The Jackson Laboratory) were used to analyze the phase of the liver circadian rhythm. Genotyping was performed by measuring light emission from fresh tail pieces using a luminometer. Male wild-type C57BL/6J mice from our breeding colony were used to assess eating behavior rhythms. All mice were bred and maintained in 12h light:12h dark (light intensity ~350 lux) with chow (13.5% kcal from fat, LabDiet 5L0D; 3.02 kcal/g metabolizable energy) and water provided *ad libitum* in the Vanderbilt University animal facility. Sentinel testing in the animal facility was performed biannually and mice were negative for all Vanderbilt excluded pathogens (excluded pathogens listed at https://www4.vanderbilt.edu/acup/dac/List_of_Excluded_Pathogens_050112.pdf; our colony is negative for mouse parvovirus). Mice were weaned at 3 weeks old and group housed (2 to 4 mice per cage). Starting at 7 weeks old, body weight and food were measured weekly (during the 3 hours before lights off).

### Ethics Statement

All experiments were conducted in accordance with the guidelines of the National Institutes of Health Guide for the Care and Use of Laboratory Animals. Mice were euthanized by cervical dislocation without anesthesia followed by decapitation. All procedures were approved by the Institutional Animal Care and Use Committee at Vanderbilt University (protocol number M/13/081).

### Experiment I. Protocol for measuring liver circadian rhythm

**Fig 1:**
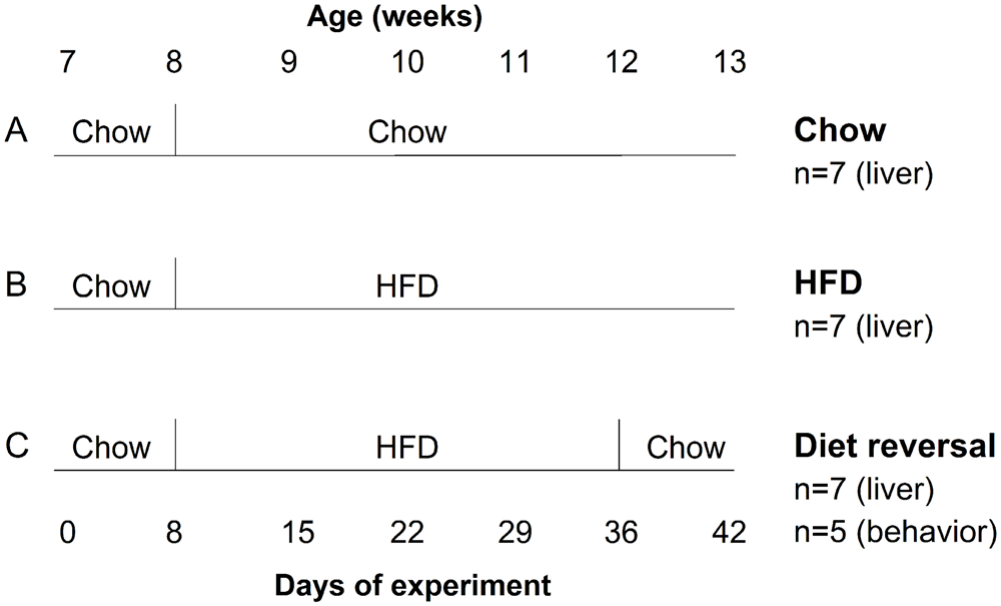
Experimental Protocol Diagram. Male heterozygous PER2::LUC mice were single-housed in light-tight boxes (12L:12D) at 7 weeks old and fed either chow for 5 weeks (A), high-fat diet (HFD) for 5 weeks (B), or HFD for 4 weeks and then chow for 1 week (C). At 13 weeks old, bioluminescence rhythms were measured from liver explants (n = 7/group). Eating and locomotor activity rhythms were also measured in the diet reversal group (C, n = 5).

At 7 weeks old, male heterozygous PER2::LUC mice were singly housed in cages (33 x 17 x 14 cm) with locked running wheels (running wheels were present but could not rotate) and fed chow *ad libitum*. The cages were housed in light-tight, ventilated boxes in 12h light:12h dark (light intensity ~200–300 lux) at 25.5±1.5°C. At 8 weeks old, mice were either fed chow for 5 weeks (Fig 1A; n = 7), fed high-fat diet (45% kcal from fat; Research Diets D01060502; 4.73 kcal/g) for 5 weeks (Fig 1B; n = 7), or fed high-fat diet for 4 weeks and then chow for 1 week (Fig 1C; n = 7). At 13 weeks old, PER2::LUC expression was measured in *ex vivo* liver cultures.

### Bioluminescence recording and analysis

Mice were euthanized by cervical dislocation followed by decapitation. For most experiments, mice were euthanized at the end of the light phase, within 1.5h before lights out. To determine if the phase of the liver PER2::LUC rhythm was reset by the culture procedure, some mice were euthanized at the beginning of the light phase, within 1.5 h of lights on. Liver explants were cultured as previously described except that CellGro (catalog number 90-013PB plus L-glutamine) recording medium was used [18]. Liver explants were cultured on mesh (Spectra Mesh Woven Filter, 500μm opening) in 35mm dishes in the LumiCycle apparatus (Actimetrics Inc, Evanston, IL) housed in a water-jacketed incubator (temperature 36.5°C ± 0.03°C). Photon counts were integrated over 10-min intervals by the LumiCycle. Bioluminescence data were detrended by subtracting the 24-h moving average and smoothed by 0.5h adjacent averaging using Lumicycle software and then exported to Clocklab analysis software (Actimetrics Inc.). The phase of the liver PER2::LUC rhythm was defined as the peak of bioluminescence occurring between 12h and 36h in culture. To determine if the phase of the liver PER2::LUC rhythm was reset by the culture procedure, cultures were prepared at either Zeitgeber time (ZT) 1 (where ZT12 is lights off) or ZT11 (~10h apart) and the peaks of bioluminescence were plotted relative to the light-dark cycle and relative to the time of culture.

### Experiment II. Protocol for measuring eating behavior and locomotor activity

The experimental conditions were identical to those described above for measuring the liver circadian rhythm except that eating behavior and locomotor activity were simultaneously recorded in one group of wild-type male C57BL/6J mice that were fed high-fat diet for 4 weeks and then chow for 1 week (Fig 1C; n = 5).

### Behavior recording and analysis

General locomotor activity data were collected every minute using passive infrared sensors (sensors record a maximum of 1 count every 6 secs; model 007.1, Visonic LTD). Double-plotted actograms of locomotor activity were created with Clocklab (10-min bins; normalized setting). Eating behavior was continuously recorded using an infrared video camera (PYLE PLCM22IR Flush Mount Rear View Camera with 0.5 lux Night Vision, Pyle Audio Inc., Brooklyn, NY) interfaced to a computer with VideoSecu4 [11]. Eating behavior was analyzed in 1-minute bins (coded as 1 for eating behavior and 0 for no eating behavior) as previously described [11]. Eating behavior data were plotted in circular histograms (Oriana 4.0; Kovach Computing Services, Wales, UK).

### Statistical Analyses

Body weights (at 13 weeks old) and the phases of liver PER2::LUC rhythms were compared by one-way ANOVA followed by post-hoc Fisher’s least significant difference (LSD) tests (OriginPro 9.1, Northampton, MA). Circular data were plotted and analyzed using Oriana 4.0. The mean vector of each day of behavior data (for individual mice) was determined by Rayleigh’s uniformity test to indicate the angle (μ) and degree of clustering (length; r). Grand mean vectors (to analyze groups of mice) were analyzed using Hotelling’s one sample test. The length of the vector describes the uniformity of the distribution of activity such that short vectors indicate that activity is more evenly distributed across the cycle. Significance was ascribed at *p*<0.05.

## Results

### Diet-induced obesity is rapidly ameliorated by diet reversal

**Fig 2:**
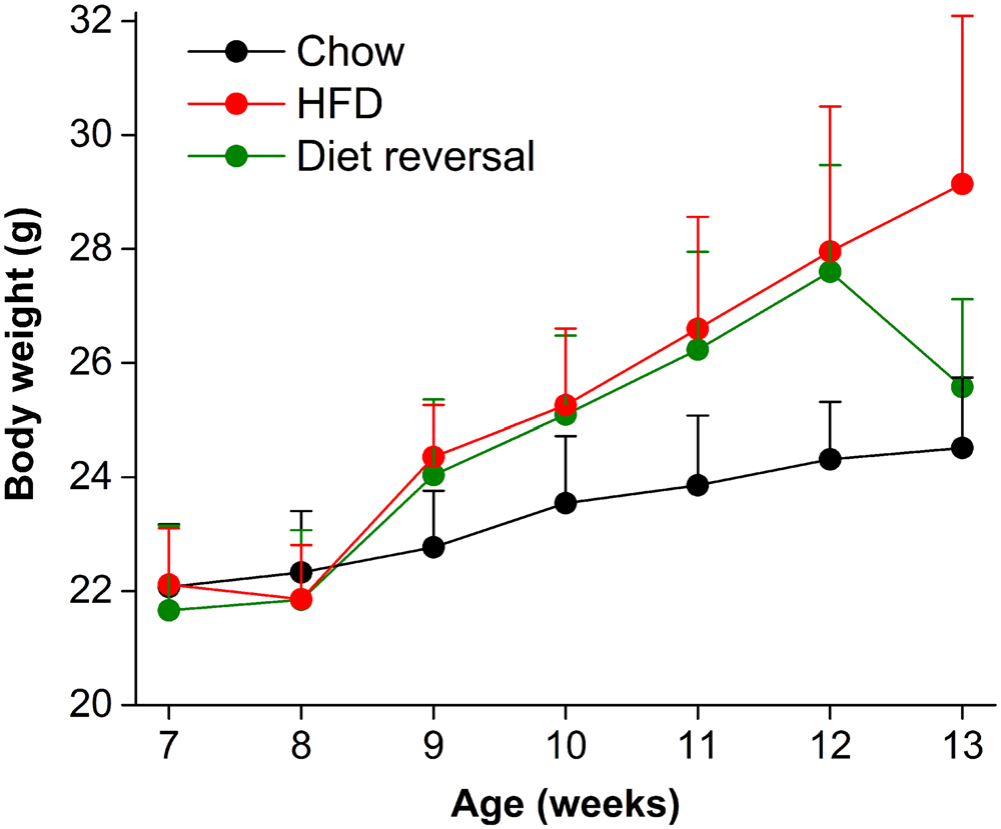
Diet-induced obesity is rapidly reversed. Weekly body weights (mean grams ± SD) of male heterozygous PER2::LUC mice fed either chow for 5 weeks (black circles), high-fat diet for 5 weeks (red circles), or high-fat diet for 4 weeks and then chow for 1 week (green circles).

After 5 weeks on high-fat diet, mice weighed significantly more than chow-fed mice (Fig 2; 13 weeks old, F_(2,23)_ = 10.01, *p*<0.001, LSD *p*<0.001). The body weight of diet reversal mice did not differ from chow-fed mice (LSD *p* = 0.24). Thus, diet-induced obesity was completely reversed by 1 week of chow feeding.

### The phase of the liver circadian rhythm is restored by diet reversal

**Fig 3:**
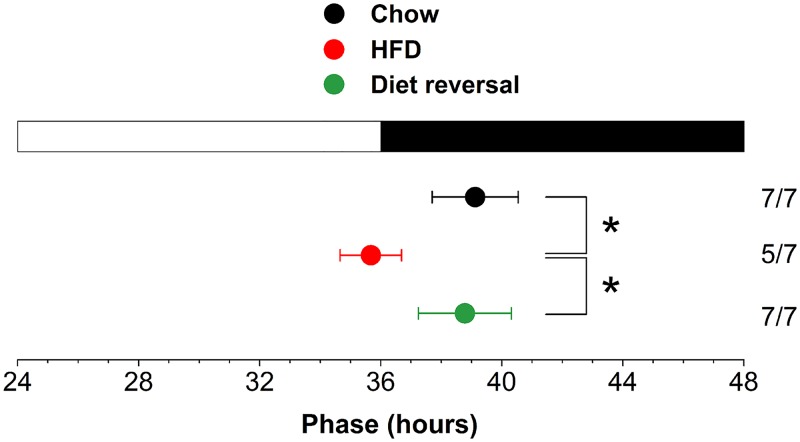
The phase of the liver clock is restored by diet reversal after chronic high-fat diet consumption. Male heterozygous PER2::LUC mice were fed either chow for 5 weeks (black circle), high-fat diet for 5 weeks (red circle), or high-fat diet for 4 weeks and then chow for 1 week (green circle). The mean (±SD) phases were determined from the peaks of PER2::LUC expression in liver explants during the interval between 12 and 36h in culture and were plotted relative to the time of last lights on (24h is lights on and 36h is lights off, white and black bar on top; liver cultures were prepared at ZT11). The sample size is shown (number of rhythmic tissues/number of tissues tested). *p<0.01.

We previously showed that the phase of the liver PER2::LUC bioluminescence rhythm was advanced ~5h after 1 week of high-fat diet consumption [11]. To determine if the liver phase remained advanced during chronic high-fat diet consumption, we fed mice high-fat diet for 5 weeks and measured PER2::LUC bioluminescence rhythms in liver explants (S1 Fig). The phase of the liver was advanced 4h after 5 weeks of high-fat diet eating compared to chow-fed mice (F_(2,18)_ = 10.51, *p*<0.01, LSD *p*<0.001; Fig 3). To determine if the phase of the liver clock was reversible, we fed mice high-fat diet for 4 weeks and then chow for 1 week. By 1 week of chow feeding, the phase of the PER2::LUC rhythm in liver did not differ from the liver rhythm in chow-fed mice (LSD *p* = 0.65). These data demonstrate that the phase of the liver rhythm was reversible even after chronic high-fat diet consumption.

To determine if the 4-h advance of the liver PER2::LUC rhythm in high-fat diet-fed mice was an artifact of the culture procedure, we prepared liver explants from chow or high-fat diet-fed mice at either ZT1 (1h after lights on) or ZT11 (1h before lights off; S2 Fig). We found that the absolute phase of the liver PER2::LUC rhythm was advanced ~2h in cultures prepared at ZT1 vs. ZT11 in both chow (S2A Fig) and high-fat diet-fed mice (S2B Fig; peak of PER2::LUC expression was plotted relative to the light-dark cycle). The liver clock was not reset by the culture procedure in chow- (S2C Fig) and high-fat diet-fed (S2D Fig) mice because the peak of PER2::LUC expression did not occur at a fixed interval after culture in explants prepared at ZT1 and ZT11. Together these data show that culture time can shift the absolute phase of the liver clock (by ~2h), but the ~4h advance caused by eating high-fat diet compared to eating chow was maintained in both the ZT1 and ZT11 culture conditions.

### The effects of chronic high-fat diet on the eating behavior rhythm are rapidly reversed

**Fig 4:**
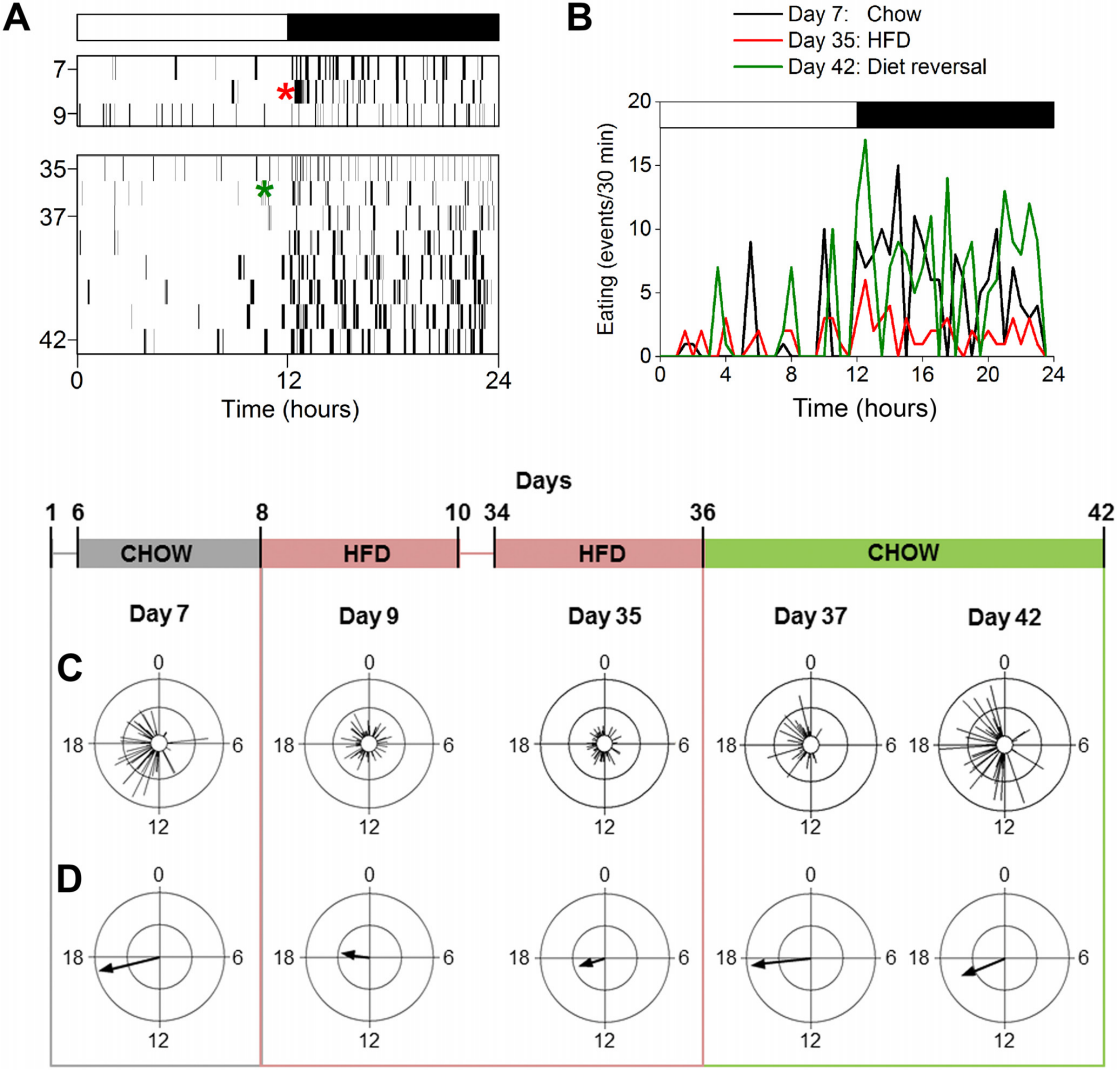
The robust eating behavior rhythm is restored immediately after diet reversal. **A.** Representative actogram of eating behavior plotted in 1-min bins (y-axis: days). Chow was replaced with high-fat diet (HFD; red asterisk) on day 8 and HFD was replaced with chow (green asterisk) on day 36. **B.** Representative activity profile (30-min bins) of eating behavior from one mouse during chow (day 7, black), chronic HFD (day 35, red), and diet reversal (day 42, green). **C.** Representative circular histograms of eating behavior (10-min bins) in an individual mouse during one day of chow (day 7), 4 weeks of HFD consumption (days 9, 35), and upon return to chow (days 37, 42). Scale: inner circle, 0; middle circle, 5; outer circle, 10. **D.** Grand mean vectors of eating behavior (n = 5). Scale: inner circle, 0; middle circle, 0.3; outer circle, 0.6. Lights were on from 0–12. Circular statistics are shown in Table 1.

Eating behavior was consolidated during the night in chow-fed mice, resulting in a robust eating behavior rhythm and long mean vector [11] (Fig 4 and S3 Fig Chow: Day 7; Table 1). On the first day of high-fat diet consumption, eating behavior was distributed across the day and night, resulting in a low-amplitude or absent eating behavior rhythm. The low-amplitude rhythm was evidenced by a short mean vector and observed in activity profiles of eating behavior of individual mice (Fig 4, HFD: Day 9; S3 Fig). The effect of high-fat diet on the distribution of eating behavior persisted for the duration of chronic feeding (Fig 4 and S3 Fig, HFD: Day 35; data from each of 4 weeks of high-fat diet shown in S4 Fig). On the first day of diet reversal, eating events were consolidated during the night and the robust eating behavior rhythm (and long vector) was restored (Fig 4, S3, S5 and S6 Figs: Day 37; Table 1). By 1 week of diet reversal, the eating behavior rhythm was similar the rhythm prior to high-fat diet consumption (Fig 4, Table 1, S3, S6 Figs). These data show that high-fat diet consumption chronically disrupted the eating behavior rhythm, but this effect was rapidly reversed by chow feeding.

**Table 1:** Mean vector properties of eating behavior rhythms.

	Day 7	Day 9	Day 35	Day 37	Day 42
**Mean angle(μ)** †	257 (234–274)	278 (206–333)	253(195–303)	263 (230–306)	248 (216–269)
**Length (r)**	0.58	0.28	0.26	0.56	0.44
***P*** *	F = 330, *p* = 3x10^-4^	F = 22, *p* = .02	F = 39, *p* = .01	F = 60, *p* = .004	F = 174, *p* = 8x10^-4^

*Hotelling’s one sample test was used to test if there was a significant mean direction.

^†^The 95% confidence intervals are reported (in parentheses) for the directions of the grand mean vectors.

### The reversibility of high-fat diet disruption of locomotor activity rhythms is variable

**Fig 5:**
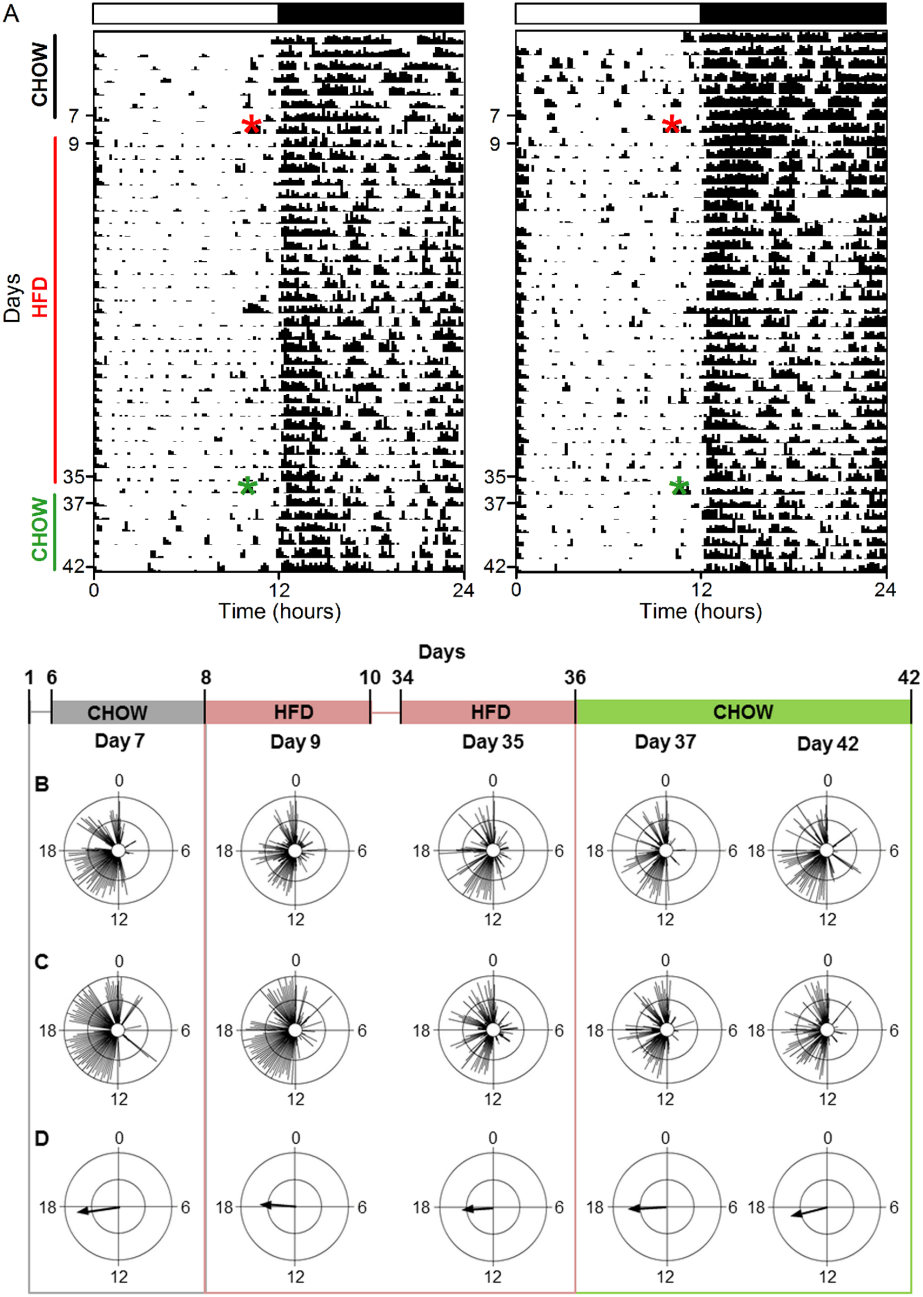
The reversibility of high-fat diet disruption of locomotor activity rhythms is variable. **A.** Representative actograms of locomotor activity plotted in 10-min bins. Chow was replaced with high-fat diet (HFD; red asterisk) on day 8 and HFD was replaced with chow (green asterisk) on day 36. **B, C.** Representative circular histograms of activity (10-min bins) in 2 individual mice during one day of chow (day 7), 4 weeks of HFD consumption (days 9, 35), and upon return to chow (days 37, 42). Scale: inner circle, 0; middle circle, 5; outer circle, 10. **C.** Grand mean vectors of locomotor activity (n = 5). Scale: inner circle, 0; middle circle, 0.3; outer circle, 0.6. Lights were on from 0–12. Circular statistics are shown in Table 2.

The locomotor activity of chow-fed mice occurred mostly during the dark phase of the light-dark cycle, with several consolidated bouts of activity during the day [11] (Fig 5A, S7 Fig: days 1–7; Fig 5B–5D, S8 and S9 Figs: day 7; Table 2). Beginning on the first day of high-fat diet and persisting for the duration of chronic high-fat feeding, activity in the light phase was dispersed across the day (instead of occurring in several distinct bouts; Fig 5A: days 9–35; Fig 5B–5D: day 9, 35; S8 and S9 Figs). High-fat diet consumption also altered activity during the dark phase; the most notable change was a reduction in nighttime activity (as in Fig 5B and 5C; S9A, S9C–S9E Fig). In general, high-fat diet caused a modest reduction in the amplitude of the locomotor activity rhythm (shortened vector length in Fig 5D: day 9, 35; Table 2). The effects of diet reversal on the activity rhythm varied between animals (Fig 5: days 37–42; all individual mice shown in S9 and S10 Figs, S1 Table). The locomotor activity rhythm was restored to normal after 2 weeks of diet reversal in 2 mice (Fig 5A, left, Fig 5B; S10A and 10B Fig: Day 49; S1 Table). In 2 mice, the daytime activity pattern was restored by 2 (S7A, S10A Figs: day 38) or 6 days (S7C, S10C Figs: day 42) of diet reversal, while 3 other mice never reverted to the normal daytime pattern (S7B, S7D, S7E, S10B, S10D, S10E Figs). The nighttime activity pattern was restored in 3 mice by 2 weeks of diet reversal (S7A, S7C, S7E, S10A, S10C, S10E Figs: day 49), but not in 1 other mouse (S7D and S10D Figs: day 49; note that nighttime activity was not altered by high-fat diet in the mouse shown in S7B Fig). Together, these data show that the daily pattern of locomotor activity was rescued by diet reversal in some mice, but not in others.

**Table 2:** Vector properties of locomotor activity rhythms in individual mice.

	Mouse ID	Day 7	Day 9	Day 35	Day 42	Day 49
**Mean angle(μ) ± SD** *	A	253±64	265±80	239±75	240±71	266±75
	B	277±81	276±76	255±89	255±88	268±75
	C	263±71	295±83	280±80	264±76	254±77
	D	272±67	268±71	280±79	270±71	266±82
	E	251±66	265±80	272±82	252±68	254±72
**Length (r)** *	A	0.53	0.38	0.43	0.46	0.43
	B	0.37	0.41	0.30	0.30	0.43
	C	0.47	0.35	0.37	0.41	0.41
	D	0.51	0.47	0.38	0.46	0.36
	E	0.52	0.38	0.36	0.49	0.45

*The mean angle (μ) ± circular standard deviation (SD) and vector length (r) are reported are for individual mice.

*Rayleigh’s Uniformity test was used to determine if the locomotor activity of individual mice had a significant non-uniform direction (all vectors were *p*<1x10^-12^).

## Discussion

We previously showed that the liver circadian clock, the daily rhythm of eating behavior, and the daily pattern of locomotor activity were acutely sensitive to high-fat diet consumption [11]. However, it was unknown whether these effects persisted during chronic high-fat diet feeding and whether they were reversible.

After chronic (5 weeks) high-fat feeding, the phase of the liver molecular clock was advanced 4h. This effect was completely reversed by 1 week of chow feeding. Thus, the liver circadian rhythm is exquisitely sensitive to macronutrients, even after chronic exposure to high-fat diet. Likewise, the temporal transcriptional state of the liver is also reversible after chronic high-fat diet consumption [19].

Importantly, we found that the 4-h advance in the phase of the liver clock caused by high-fat diet consumption was not an artifact of the culture procedure. The phase of the liver PER2::LUC rhythm was advanced ~2h when the liver was cultured in the morning compared to livers cultured in the evening, but this occurred in both chow- and high-fat fed mice. Moreover, the phases of ex vivo liver PER2::LUC rhythms cultured from chow-fed mice (at either time of day) approximated the phase of the PERIOD2 rhythm measured in vivo [20]. Therefore, our results reflect the effects of diet on the phase of the liver PER2::LUC rhythm and were not an artifact of the experimental approach.

This study also demonstrated that the phase of the liver clock correlated with body weight. The phase of the liver circadian clock was advanced during weight gain (positive energy balance) but returned to its normal phase during weight loss and weight maintenance. While it is unknown whether the change in the phase of the liver clock precedes or is a consequence of changes in energy balance, it is intriguing to speculate that the phase of the liver circadian clock contributes to the metabolic dysfunction caused by high-fat diet consumption.

The daily rhythm of eating behavior was markedly disrupted during chronic high-fat feeding. The rhythm was severely compromised (or absent) on the first day of high-fat eating, such that mouse eating behavior was almost evenly distributed across the entire 24h day [11]. In this study, we demonstrated that the effect of high-fat diet persisted during chronic high-fat diet consumption. Beginning on day 1 of high-fat diet and persisting for the entire 4 week feeding protocol, the amplitude of the eating behavior rhythm was markedly reduced or the rhythm was absent. This is consistent with the study by Kohsaka et al. (2007) that showed compromised food intake rhythms during 6 weeks of high-fat feeding. For the first time, we showed that the effects of high-fat diet consumption on the daily rhythm of eating behavior were rapidly reversed. Within 2 days of chow feeding (after 4 weeks of high-fat diet feeding), the eating behavior rhythm was similar to the rhythm prior to high-fat feeding.

Chronic high-fat diet consumption also altered the pattern of locomotor activity. By 1 day of high-fat feeding, locomotor activity became dispersed across the daytime and activity was decreased during the nighttime. Unlike eating behavior, locomotor activity was not immediately restored by diet reversal. In fact, the locomotor activity of some mice never reverted during the 2 weeks of diet reversal examined in this study. Because the locomotor activity rhythm is controlled by the SCN, it is possible that the SCN rhythm is altered by high-fat diet consumption. We think this is unlikely since in our previous study we found that the locomotor activity rhythm was altered on the first day of high-fat diet consumption, yet there was no effect on the phase, period, and amplitude of the SCN PER2::LUC rhythm [11]. Instead, we speculate that a brain region distinct from the SCN responds to high-fat diet which results in abnormal locomotor activity patterns.

In sum, we found that high-fat diet had long-lasting effects on the liver clock, daily rhythm of eating behavior, and daily pattern of locomotor activity. Upon diet reversal, the eating behavior rhythm was rapidly reversed (within 2 days) and the phase of the liver clock was rescued by 7 days of diet reversal. In contrast, some characteristics of the daily pattern of locomotor activity were not restored after 2 weeks of diet reversal.

Together these data demonstrate that the circadian system is sensitive to changes in macronutrient composition of food. The presence of obesity does not inhibit the sensitivity of the liver circadian clock and the brain region(s) controlling the daily rhythm of eating behavior (in an unidentified anatomical locus) to changes in the fat content of food as even obese mice experienced a complete reversal of the effects of high-fat diet on these clocks. In contrast, chronic high-fat diet consumption has long-lasting effects on locomotor activity that are not quickly reversed. Thus, the liver circadian clock and eating rhythms may be ideal targets for therapeutic interventions for obesity since they are rapidly reversed even in obese animals.

## Supporting Information

S1 Fig Effects of high-fat diet and diet reversal on liver bioluminescence rhythms. Detrended bioluminescence (y-axis: counts/s) recorded from liver explants prepared at ZT11 from male heterozygous PER2::LUC mice fed either chow (A), HFD for 5 weeks (B), or HFD for 4 weeks and then chow for 1 week (diet reversal; C). The light-dark cycle on the day of culture is indicated. The phases of the liver bioluminescence rhythms shown in Fig 3 were determined from the peaks of PER2::LUC expression in liver explants during the interval between 12 and 36h in culture.
(TIF)

S2 Fig The high-fat diet-induced advance of the liver PER2::LUC rhythm is not an artifact of the culture procedure. Liver explants from male heterozygous PER2::LUC mice fed either chow (A, C) or HFD (B, D) for 5 weeks were prepared at either ZT1 [AM, gray (n = 6/6) and pink (n = 4/5)] or ZT11 [PM, black (n = 6/6) and red (n = 5/7)] and bioluminescence was recorded. The phases (the peaks between 12-36h in vitro) of PER2::LUC rhythms from individual mice were plotted relative to the light-dark (LD) cycle (A, B) or relative to the time of culture (C, D). The ZT11 HFD (red) data are the same data as in Fig 3.
(TIF)

S3 Fig Daily distribution of eating behavior during chow, chronic high-fat diet consumption, and diet reversal. Activity profiles (left column; 30-min bins) of eating behavior from individual mice (A-E) during chow (day 7, black), chronic high-fat diet consumption (day 35: red), and diet reversal (day 42, green). C is shown in Fig 4B. Distribution of eating events in the 12h light phase (white bars) and 12h dark phase (black bars) of the day in individual mice during chow, high-fat diet, and diet reversal (right column).
(TIF)

S4 Fig Daily rhythms of eating behavior during chronic high-fat diet consumption. **A.** Representative circular histograms of eating behavior (10-min bins) in an individual mouse. Male wild-type mice were fed high-fat diet for 4 weeks and eating behavior was analyzed 1 day of each week. Scale: inner circle, 0; middle circle, 5; outer circle, 10. **B.** Grand mean vectors of eating behavior (n = 5). Scale: inner circle, 0; middle circle, 0.3; outer circle, 0.6. Lights were on from 0–12.
(TIF)

S5 Fig Eating behavior during diet reversal. Actograms (y-axis: days) of eating behavior of 5 individual mice (A-E) plotted in 1-min bins. Chow was replaced with high-fat diet (HFD; red asterisk) on day 8 and HFD was replaced with chow (green asterisk) on day 36. C is shown in Fig 4A.
(TIF)

S6 Fig Circular histograms of daily eating behavior during diet reversal. **A.** Representative circular histograms of eating behavior (10-min bins) in an individual mouse during diet reversal. Male wild-type mice were fed high-fat diet for 4 weeks and were returned to chow diet on day 36. Scale: inner circle, 0; middle circle, 5; outer circle, 10. **B.** Grand mean vectors of eating behavior (n = 5). Scale: inner circle, 0; middle circle, 0.3; outer circle, 0.6. Lights were on from 0–12.
(TIF)

S7 Fig Locomotor activity rhythms during chronic high-fat diet consumption and diet reversal. Actograms of locomotor activity in individual mice (A-E) plotted in 10-min bins (normalized setting in Clocklab). Chow was replaced with high-fat diet (HFD; red asterisk) on day 8 and HFD was replace with chow (green asterisk) on day 36. A and D are shown in Fig 5A.
(TIF)

S8 Fig Daily rhythms of locomotor activity during chronic high-fat diet consumption. Circular histograms of locomotor activity (10-min bins) in individual mice (A-E). Male wild-type mice were fed chow (days 1–7) and then high-fat diet for 4 weeks (days 9–35). Scale: inner circle, 0; middle circle, 5; outer circle, 10. Lights were on from 0–12.
(TIF)

S9 Fig Daily distribution of locomotor activity during chow, chronic high-fat diet consumption, and diet reversal. Activity profiles (left column; 30-min bins) of locomotor activity measured by passive infrared sensors from individual mice (A-E) during chow (day 7, black), chronic high-fat diet consumption (day 35: red), and diet reversal (day 42, green). Distribution of locomotor activity in the 12h light phase (white bars) and 12h dark phase (black bars) of the day in individual mice during chow, high-fat diet, and diet reversal (right column).
(TIF)

S10 Fig Daily rhythms of locomotor activity during diet reversal. Circular histograms of locomotor activity (10-min bins) in individual mice (A-E) during diet reversal. Male wild-type mice were fed high-fat diet for 4 weeks and were returned to chow diet on day 36. Scale: inner circle, 0; middle circle, 5; outer circle, 10. Lights were on from 0–12.
(TIF)

S1 File Body weight and liver phase data. (XLSX)

S2 File Eating behavior raw data. (XLSX)

S3 File ARRIVE Guidelines checklist. (PDF)

S1 Table Vector properties of locomotor activity rhythms in individual mice during diet reversal. (PDF)
