# Supplementary figures and images for: Disruption of Daily Rhythms by High-Fat Diet Is Reversible
Source: PLoS One. 2015 Sep 14;10(9):e0137970. doi: 10.1371/journal.pone.0137970 (PMC4569368; doi:10.1371/journal.pone.0137970)

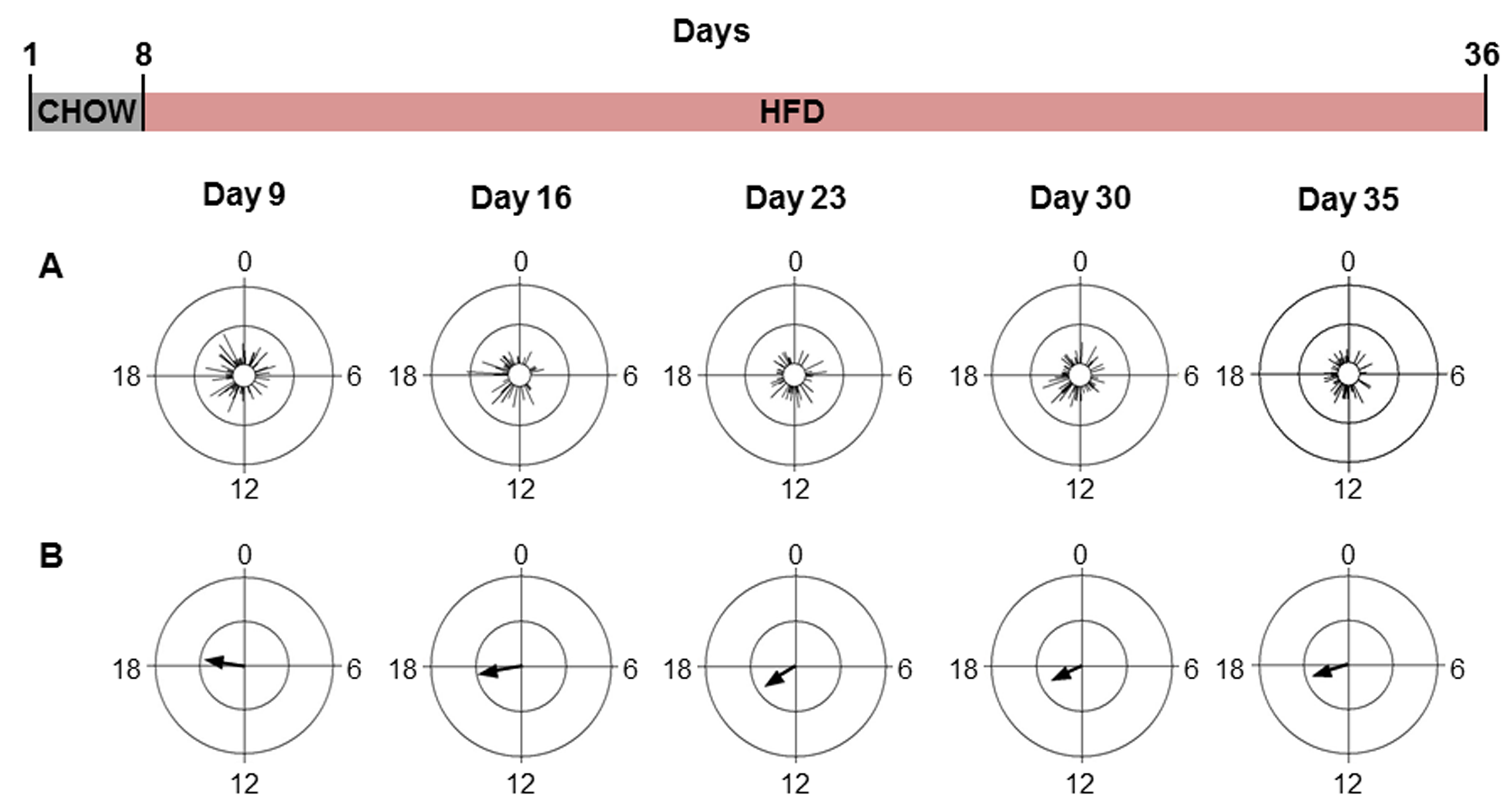

Supplement: S4 Fig — A. Representative circular histograms of eating behavior (10-min bins) in an individual mouse. Male wild-type mice were fed high-fat diet for 4 weeks and eating behavior was analyzed 1 day of each week. Scale: inner circle, 0; middle circle, 5; outer circle, 10. B. Grand mean vectors of eating behavior (n = 5). Scale: inner circle, 0; middle circle, 0.3; outer circle, 0.6. Lights were on from 0–12. (TIF) [file pone.0137970.s004.tif]

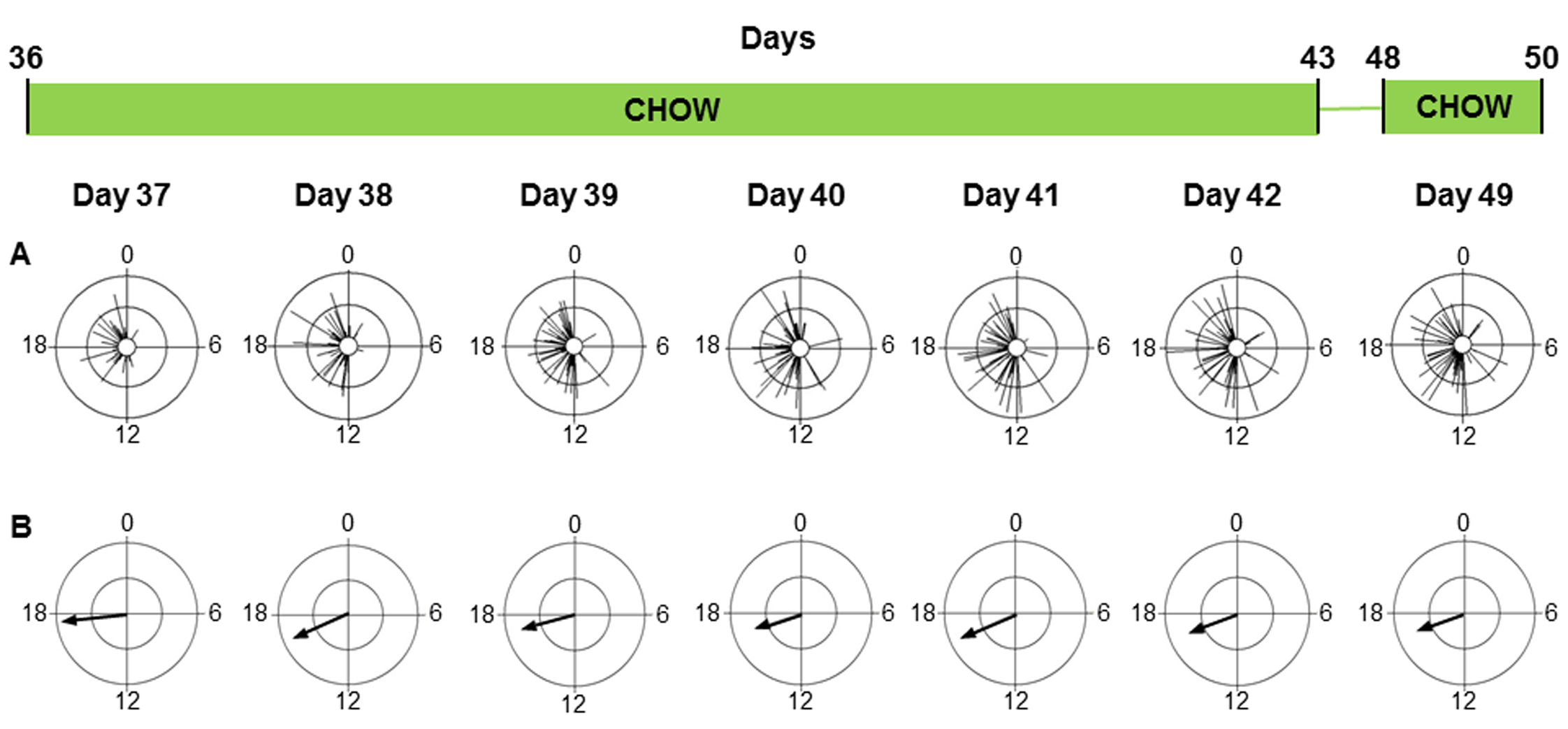

Supplement: S6 Fig — A. Representative circular histograms of eating behavior (10-min bins) in an individual mouse during diet reversal. Male wild-type mice were fed high-fat diet for 4 weeks and were returned to chow diet on day 36. Scale: inner circle, 0; middle circle, 5; outer circle, 10. B. Grand mean vectors of eating behavior (n = 5). Scale: inner circle, 0; middle circle, 0.3; outer circle, 0.6. Lights were on from 0–12. (TIF) [file pone.0137970.s006.tif]

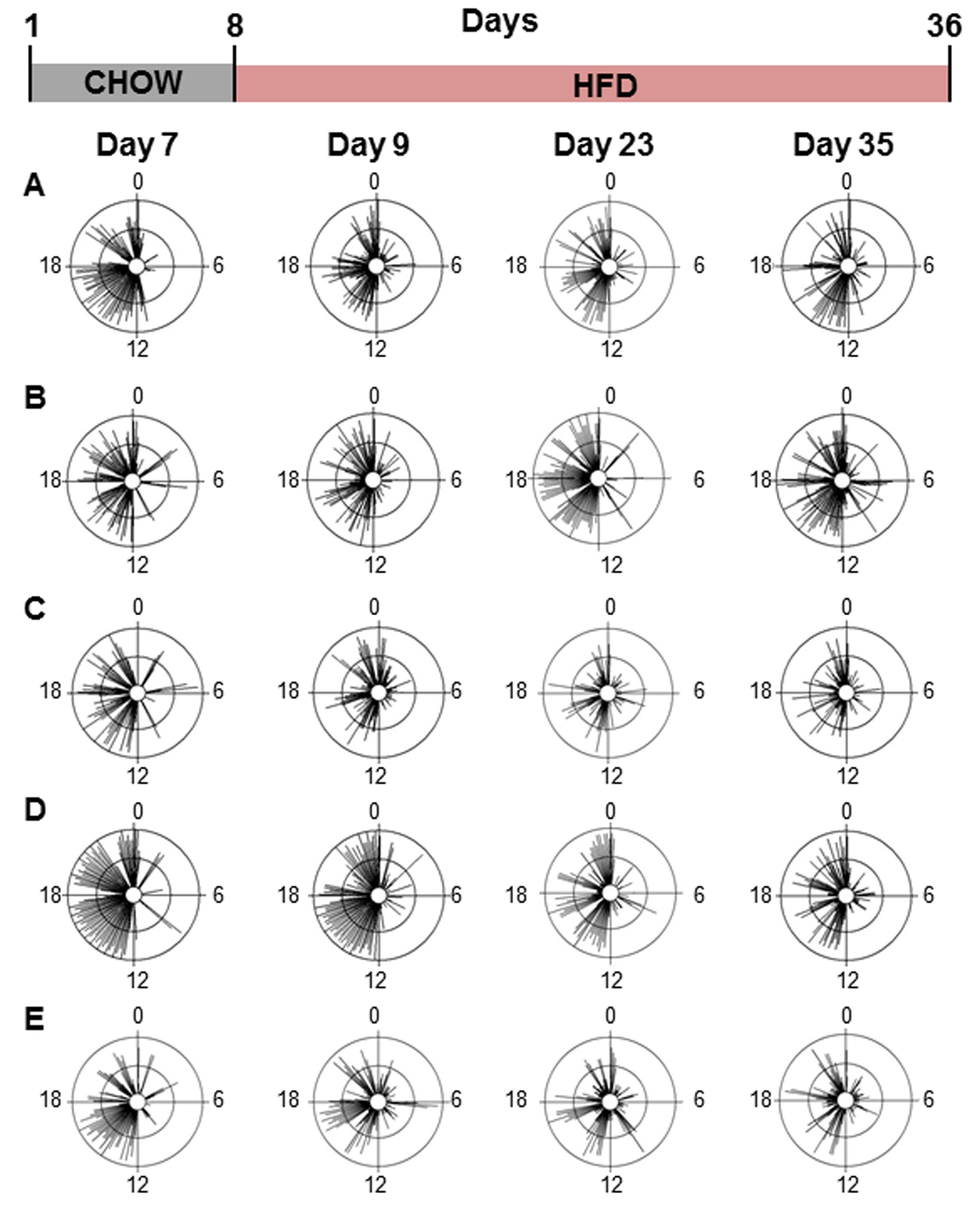

Supplement: S8 Fig — Circular histograms of locomotor activity (10-min bins) in individual mice (A-E). Male wild-type mice were fed chow (days 1–7) and then high-fat diet for 4 weeks (days 9–35). Scale: inner circle, 0; middle circle, 5; outer circle, 10. Lights were on from 0–12. (TIF) [file pone.0137970.s008.tif]

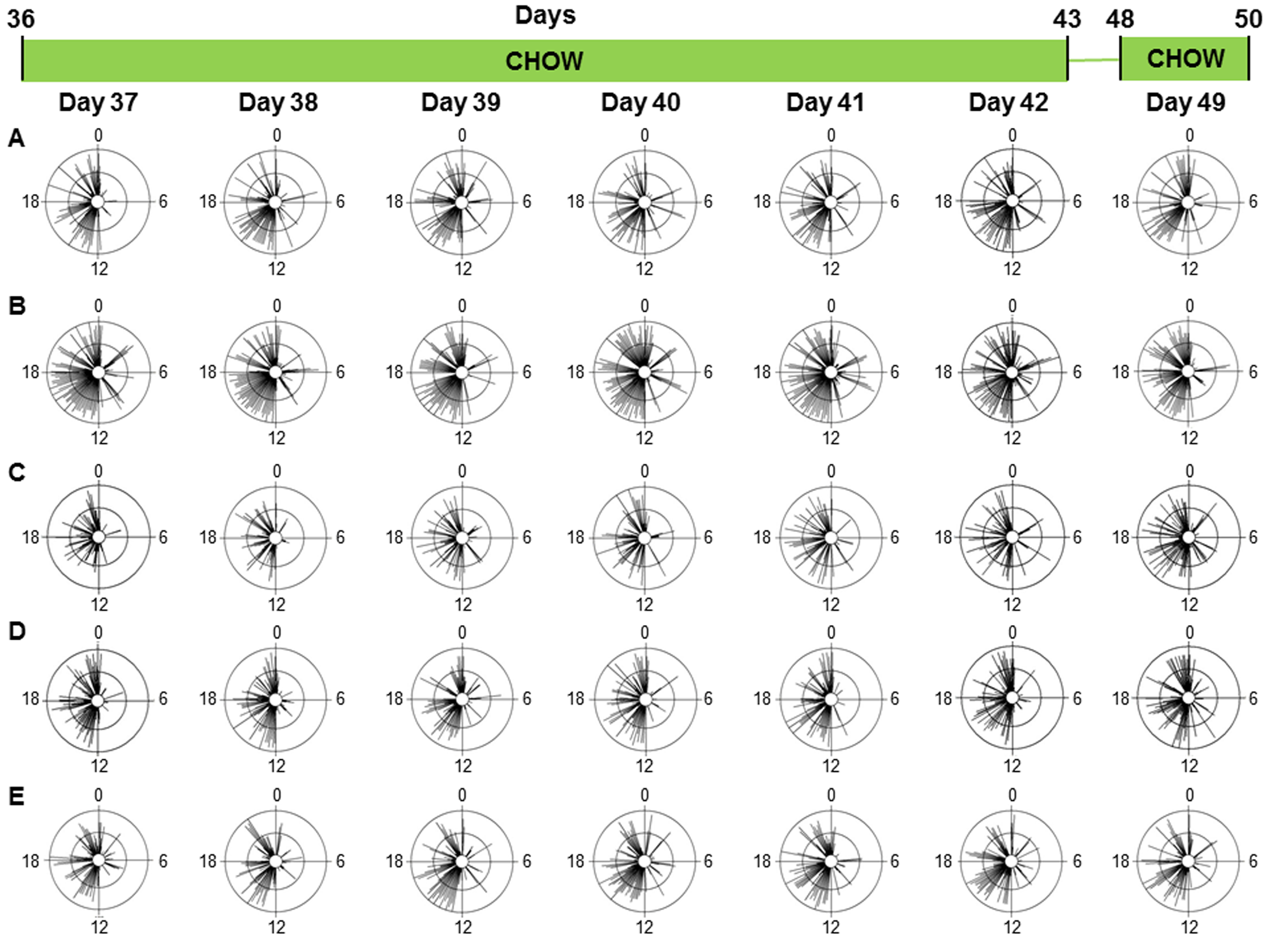

Supplement: S10 Fig — Circular histograms of locomotor activity (10-min bins) in individual mice (A-E) during diet reversal. Male wild-type mice were fed high-fat diet for 4 weeks and were returned to chow diet on day 36. Scale: inner circle, 0; middle circle, 5; outer circle, 10. Lights were on from 0–12. (TIF) [file pone.0137970.s010.tif]
