# Supplementary material for: Disruption of Daily Rhythms by High-Fat Diet Is Reversible
Source: PLoS One. 2015 Sep 14;10(9):e0137970. doi: 10.1371/journal.pone.0137970 (PMC4569368; doi:10.1371/journal.pone.0137970)
Supplement: S1 Table — (PDF) [file pone.0137970.s014.pdf]

S1 Table. Vector properties of locomotor activity rhythms in individual mice during diet reversal.

	Mouse ID	Day 37	Day 38	Day 39	Day 40	Day 41	Day 42	Day 49
**Mean Angle (μ) ± SD***	**A**	260±72	236±68	253±78	240±77	248±70	240±71	266±75
	**B**	257±76	252±75	262±72	271±86	253±85	255±88	268±75
	**C**	279±69	268±67	258±74	269±75	261±68	264±76	254±77
	**D**	270±70	257±73	260±79	268±73	264±69	270±73	266±82
	**E**	265±79	256±70	244±68	250±74	252±72	252±68	254±72
**Length (r)***	**A**	0.45	0.49	0.40	0.40	0.48	0.46	0.43
	**B**	0.41	0.43	0.45	0.33	0.33	0.30	0.43
	**C**	0.49	0.51	0.43	0.42	0.49	0.41	0.41
	**D**	0.47	0.44	0.38	0.45	0.48	0.46	0.36
	**E**	0.39	0.47	0.49	0.43	0.46	0.49	0.45

*The mean angle (μ) ± circular standard deviation (SD) and vector length (r) are reported are for individual mice. *Rayleigh's Uniformity test was used to determine if the locomotor activity of individual mice had a significant non-uniform direction (all vectors were $p<1\text{x}10^{-12}$).
